# Impact of COPD on COVID-19 prognosis: A nationwide population-based study in South Korea

**DOI:** 10.1038/s41598-021-83226-9

**Published:** 2021-02-12

**Authors:** Sang Chul Lee, Kang Ju Son, Chang Hoon Han, Seon Cheol Park, Ji Ye Jung

**Affiliations:** 1 Division of Pulmonology, Department of Internal Medicine, National Health Insurance Service Ilsan Hospital, 100 Ilsan-ro, Ilsandong-gu, Goyang, 10444 Republic of Korea; 2 Graduate School, Yonsei University College of Medicine, Seoul, Republic of Korea; 3 Department of Research and Analysis, National Health Insurance Service Ilsan Hospital, Goyang, Republic of Korea; 4 Department of Biostatistics and Computing, Yonsei University Graduate School, Seoul, Republic of Korea; 5 Division of Pulmonary and Critical Care Medicine, Department of Internal Medicine, Severance Hospital, Yonsei University College of Medicine, 50-1 Yonsei-ro, Seodaemun-gu, Seoul, 03722 Republic of Korea

**Keywords:** Chronic obstructive pulmonary disease, Viral infection, Risk factors

## Abstract

Underlying chronic respiratory disease may be associated with the severity of coronavirus disease 2019 (COVID-19). This study investigated the impact of chronic obstructive pulmonary disease (COPD) on the risk for respiratory failure and mortality in COVID-19 patients. A nationwide retrospective cohort study was conducted in 4610 patients (≥ 40 years old) infected with COVID-19 between January 20 and May 27, 2020, using data from the Ministry of Health and Welfare and Health Insurance Review and Assessment Service in Korea. The clinical course and various clinical features were compared between COPD and non-COPD patients, and the risks of respiratory failure and all-cause mortality in COPD patients were analyzed using a multivariate logistic regression model. Among 4610 COVID-19 patients, 4469 (96.9%) and 141 (3.1%) were categorized into the non-COPD and COPD groups, respectively. The COPD group had greater proportions of older (≥ 60 years old) (78.0% vs. 45.2%, *P* < 0.001) and male (52.5% vs. 36.6%, *P* < 0.001) patients than the non-COPD group. Relatively greater proportions of patients with COPD received intensive critical care (7.1% vs. 3.7%, *P* = 0.041) and mechanical ventilation (5.7% vs. 2.4%, *P* = 0.015). Multivariate analyses showed that COPD was not a risk factor for respiratory failure but was a significant independent risk factor for all-cause mortality (OR = 1.80, 95% CI 1.11–2.93) after adjustment for age, sex, and Charlson Comorbidity Index score. Among COVID-19 patients, relatively greater proportions of patients with COPD received mechanical ventilation and intensive critical care. COPD is an independent risk factor for all-cause mortality in COVID-19 patients in Korea.

## Introduction

Coronavirus disease 2019 (COVID-19), which is caused by severe acute respiratory syndrome coronavirus 2 (SARS-CoV-2), has caused a pandemic, with considerable morbidity and mortality^1^. The severity of disease ranges from asymptomatic infections to mild self-limiting upper respiratory tract illness, severe pneumonia with respiratory failure, or death^2^. Evidence and experience regarding treatment for COVID-19 are lacking, although comorbidities are important factors influencing patient prognosis.

Patients with chronic respiratory diseases, particularly chronic obstructive pulmonary disease (COPD), have a high risk for COVID-19 infection due to their poor underlying lung reserve and increased expression of angiotensin-converting enzyme 2 (ACE-2) receptor in the small airways^3^. However, comprehensive analyses of the risks, disease severity, and clinical course in COVID-19 patients with COPD are lacking^1,4–6^. Guan et al. evaluated the risk for serious adverse outcomes in COVID-19 patients in China by stratifying them according to the number of comorbidities^4^. A greater number of comorbidities was correlated with worse clinical outcomes, and COPD patients had the highest hazard ratio (2.68) for admission to the ICU, invasive ventilation, or death among the patients with various types of chronic underlying diseases^4^.

However, previous studies have had important limitations, with relatively small sample sizes and data obtained from a single center or region. Moreover, the impact of COPD on morbidity and mortality was not assessed in the context of other demographic factors, such as age, sex, or other comorbidities. In addition, the diagnosis and definition of COPD has not been clearly stated in previous reports^1,4–6^.

Therefore, we conducted a nationwide population-based study to analyze the impact of COPD on the risks of disease progression and mortality among COVID-19 patients in South Korea.

## Methods

### Study design

We conducted a retrospective cohort study to evaluate the risk for respiratory failure and mortality in COVID-19 patients with COPD using data from the Ministry of Health and Welfare and the Health Insurance Review and Assessment Service (HIRA) in South Korea. National Health Insurance (NHI) in Korea is a compulsory social insurance system and insures about 97% of the population. The remaining population is covered by Medical Aid. All hospitals and clinics in Korea submit medical records of patients covered by NHI and Medical Aid to the Health Insurance Review and Assessment (HIRA) office for review to be reimbursed for any healthcare services provided^7^. The HIRA claim database includes 46 million patients per year, approximately 90% of the total population in Korea, from almost 80,000 healthcare service provides^8^. The HIRA dataset comprises all insurance benefit claims by medical service providers and includes general sociodemographic information, diagnoses according to the 10th revision of the International Statistical Classification of Diseases and Related Health Problems (ICD-10), the medical institution, medications prescribed, medical costs and survival status^9^. The HIRA shared nationwide data on 7590 patients infected with COVID-19 between January 20 (date of the first confirmed case) and May 27, 2020, with researchers in South Korea.

**Figure 1:**
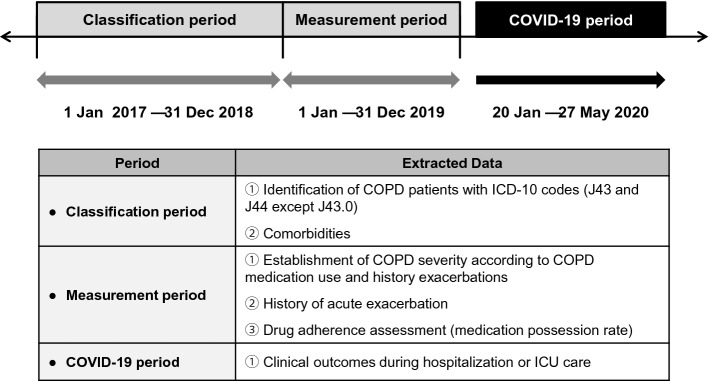
Study scheme. Information on the study population was collected for three different periods: enrollment, measurement, and COVID-19. COPD patients were identified, and the baseline characteristics of patients were assessed during the enrollment period. COPD severity, the number of acute exacerbations, and the medication possession ratio were evaluated during the measurement period. COVID-related clinical outcomes were evaluated during the COVID-19 period. COVID-19, coronavirus disease 2019; COPD, chronic obstructive pulmonary disease; ICD-10, 10th revision of the International Statistical Classification of Diseases and Related Health Problems; ICU, intensive care unit.

The present study covered three different time periods: the classification period, measurement period, and COVID-19 period (Fig. 1). Among 7590 COVID-19 patients, 4610 COVID-19 patients aged 40 years or older were categorized into the COPD and non-COPD groups according to their history of COPD diagnosis and treatment, and their comorbidities were assessed during the classification period (January 1, 2017–December 31, 2018). During the measurement period (January 1–December 31, 2019), COPD severity, the number of acute exacerbations, and drug adherence were evaluated. During the COVID-19 period, information on sex, age, region of residence, and medical aid was collected at the time of COVID-19 diagnosis. Subsequently, various clinical parameters and outcomes, such as length of hospital stay, overall medical expenses, number of intensive care unit (ICU) admissions, length of ICU stay(s), respiratory failure, and mortality, were evaluated and compared between those with and without COPD.

### Study subjects

HIRA released, via an online portal (accessible at https://hira-covid19.net), de-identified records for 7590 patients who were confirmed to be infected with COVID-19 by a reverse transcription polymerase chain reaction test for SARS-CoV-2 using nasopharyngeal swab or sputum specimens^10^. Further data linked to all of the claim records of these COVID-19 patients since January 2017 were provided from the HIRA database.

COPD was defined by the prescription of COPD medication(s) at least two times per year with a diagnosis of COPD (ICD-10 code: J43 and J44 except J43.0) during the enrollment period. The COPD medications included long-acting muscarinic antagonists (LAMAs), long-acting beta-2 agonists (LABAs), a combination of LAMA and LABA (LAMA/LABA), a combination of inhaled corticosteroid (ICS) and LABA (ICS/LABA), short-acting muscarinic antagonists (SAMAs), short-acting beta-2 agonists (SABAs), phosphodiesterase-4 (PDE-4) inhibitors, systemic beta agonists, and methylxanthine. Once identified, 141 COPD patients were compared to 4469 non-COPD patients, all of whom had confirmed cases of COVID-19.

### Definitions

The Charlson Comorbidity Index (CCI), which is used to identify the risk for mortality or patients’ resource use based on comorbidities, was recorded as previously described^11,12^. The CCI was calculated by summing the points for each relevant comorbidity, obtained based on diagnostic codes in the HIRA data during the enrollment period (Supplementary Table 1).

COPD severity was assessed according to the types of medications prescribed and the number of exacerbations during the measurement period. Then, COPD patients were categorized into severe and non-severe groups. The group with severe disease included those who had experienced exacerbations two or more times and those who had been prescribed triple therapy (ICS, LABA, and LAMA), PDE-4 inhibitors, or low-dose macrolides.

An acute exacerbation was defined when the diagnostic code for COPD (J43 and J44 except J43.0) was present in conjunction with any of the following: treatment with an antibiotic or systemic corticosteroids, hospitalization, and/or an emergency room visit^13^.

Drug adherence was determined using the medication possession rate (MPR) for COPD medications. The MPR was calculated as the sum of all days’ supplies of prescriptions filled divided by the time from the first filling of a prescription until the end of the measurement period. The patients were categorized into three adherence groups: low (MPR < 0.5), partial (MPR 0.5–0.79), and complete (MPR ≥ 0.8) adherence^14^. The previously listed COPD medications were used to measure MPR status.

Respiratory failure was defined as the use of invasive or noninvasive mechanical ventilation or extracorporeal membrane oxygenation (ECMO).

### Statistical analyses

We compared COVID-19 patients with and without COPD using χ^2^ test or Fisher exact test for categorical variables and Student’s t-test or Mann–Whitney U-test for continuous variables as indicated. To identify risk factors independently associated with the COVID-19-related clinical outcomes of respiratory failure and mortality, we conducted multivariate analyses using a logistic regression model. Risk factors are reported as adjusted odds ratios (ORs) with 95% confidence intervals (CIs). A *P* value < 0.05 was considered statistically significant. All statistical analyses were performed with SAS, version 9.4 (SAS Institute, Cary, NC, USA).

### Ethics approval

The Institutional Review Board of Severance Hospital approved the study protocol in accordance with guidelines of the Declaration of Helsinki (IRB No.: 4–2020–0248) and waived the need to obtain informed consent from the patients due to the retrospective nature of the study and the use of a de-identified database. All personal identification information was anonymized.

## Results

### Patient characteristics

**Figure 2:**
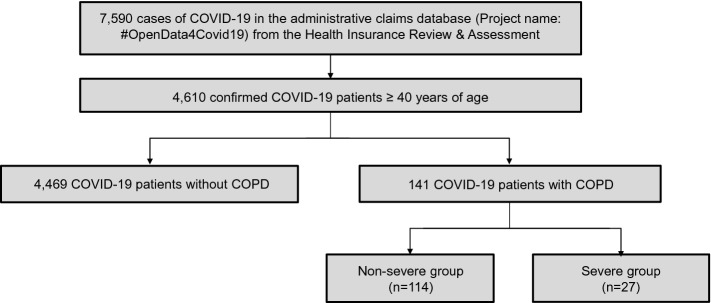
Flowchart of the study cohort. Of 7590 COVID-19 patients, 4610 were aged 40 years or older. During the enrollment period, 4469 and 141 patients were categorized into the non-COPD and COPD groups, respectively. Among the COPD patients, 114 and 27 were classified into the non-severe and severe groups, respectively. COPD was defined by the ICD-10 codes and COPD medications prescribed during the enrollment period. The severity of COPD was classified based on the types of medications prescribed and the number of exacerbations during the measurement period. COVID-19 coronavirus disease 2019; COPD, chronic obstructive pulmonary disease; ICD-10, 10th revision of the International Statistical Classification of Diseases and Related Health Problems.

Among 7590 patients infected with COVID-19 between January 20 and May 27, 2020, 4610 were aged 40 years or older. During the classification period, 4469 (96.9%) patients and 141 (3.1%) patients were categorized into the non-COPD and COPD groups, respectively. Among the COPD patients, 114 (80.9%) and 27 (19.1%) were classified into the non-severe and severe groups, respectively (Fig. 2).

The baseline demographics of the study population are shown in Table 1. The COPD group had greater proportions of older (≥ 60 years old) (78.0% vs. 45.2%, *P* < 0.001) and male (52.5% vs. 36.6%, *P* < 0.001) patients than the non-COPD group. Most comorbidities were more frequently observed in the COPD group than in the non-COPD group, and hypertension was the most common comorbidity in both groups. The proportion of patients with a CCI score of 4 or higher was 61.7% in the COPD group and 19.4% in the non-COPD group (*P* < 0.001). In the COPD group, 72.3% and 24.1% of the patients had low and complete MPRs, respectively. Moreover, 73.1% of the COPD patients experienced no moderate to severe exacerbations during the measurement period before contracting COVID-19; 12.8% experienced exacerbations two or more times.

**Table 1:** Patient characteristics.

Characteristics	Non-COPD (n = 4469)	COPD (n = 141)	*P*-value
**Age, years**			
40–59	2447 (54.8)	31 (22.0)	< 0.001
≥ 60	2022 (45.2)	110 (78.0)	
Sex, male	1636 (36.6)	74 (52.5)	< 0.001
**Comorbidities**			
Hypertension	1432 (32.0)	85 (60.3)	< 0.001
Diabetes	1110 (24.8)	74 (52.5)	< 0.001
Ischemic heart disease	309 (6.9)	33 (23.4)	< 0.001
Angina pectoris	286 (6.4)	28 (19.9)	< 0.001
Myocardial infarction	62 (1.4)	8 (5.7)	< 0.001
Heart failure	232 (5.2)	39 (27.7)	< 0.001
Cerebrovascular disease	450 (10.1)	38 (26.9)	< 0.001
Rheumatological disease	251 (5.6)	12 (8.5)	0.145
Liver disease	229 (5.1)	11 (7.8)	0.159
Malignancies	276 (6.2)	20 (14.2)	< 0.001
**CCI score**			
0–1	2354 (52.7)	13 (9.2)	< 0.001
2	739 (16.5)	30 (21.3)	
3	511 (11.4)	11 (7.8)	
≥ 4	865 (19.4)	87 (61.7)	
MPR group*			
Low	–	102 (72.3)	< 0.001
Partial	–	5 (3.6)	
Complete	–	34 (24.1)	
**Number of exacerbations**^**†**^			
0	–	103 (73.1)	< 0.001
1	–	20 (14.2)	
≥ 2	–	18 (12.8)	

Data are presented as numbers (%).

*COPD* chronic obstructive pulmonary disease, *CCI* Charlson comorbidity index, *MPR* medication possession ratio.

*Data are only for COPD patients. MPR was calculated as the sum of all days’ supplies of prescriptions filled divided by the time from the first filling of a prescription until the end of the measurement period. Patients were categorized into three adherence groups: low (MPR < 0.5), partial (MPR 0.5–0.79), and complete (MPR ≥ 0.8) adherence.

^†^Data are only for COPD patients. Exacerbation was defined when the diagnostic code for COPD (J43 and J44 except J43.0) was present in conjunction with any of the following: (1) treatment with an antibiotic or systemic corticosteroids, (2) hospitalization, or (3) an emergency room visit.

### Clinical outcomes of COVID-19 in COPD and non-COPD patients

Table 2 compares the clinical outcomes between the non-COPD and COPD groups. The rate of hospitalization, duration of hospitalization, and total amount of COVID-19-related medical resources did not differ between the two groups. However, the proportions of patients who received ICU care (7.1% vs. 3.7%, *P* = 0.041) and mechanical ventilation (5.7% vs. 2.4%, *P* = 0.015) were higher in the COPD group, although none of the COPD patients received ECMO. In addition, all-cause mortality was higher in the COPD group than in the non-COPD group (19.2% vs. 4.5%, *P* < 0.001).

**Table 2:** Clinical outcomes of COVID-19 in COPD and non-COPD patients.

Variables	Non-COPD (n = 4469)	COPD (n = 141)	*P*-value
Total amount of medical resource use, USD	5205 ± 6832	5751 ± 6720	0.349
**Hospital admission**			
Number of patients	4169 (93.3)	134 (95.0)	0.412
Length of stay, days	24.2 ± 14.9	25.9 ± 19.1	0.304
**ICU care**			
Number of patients	167 (3.7)	10 (7.1)	0.041
Length of stay, days	10.8 ± 9.0	9.7 ± 8.8	0.699
Mechanical ventilation	108 (2.4)	8 (5.7)	0.015
ECMO	21 (0.5)	0 (0)	0.415
All-cause mortality	199 (4.5)	27 (19.2)	< 0.001

Data are presented as numbers (%) or means ± standard deviations.

*COVID-19* coronavirus disease 2019, *COPD* chronic obstructive pulmonary disease, *USD* United States dollar, *ICU* intensive care unit, *ECMO* extracorporeal membrane oxygenation.

### Risk factors for respiratory failure and all-cause mortality in COVID-19 patients

Table 3 shows multivariate analyses of risk factors for respiratory failure and all-cause mortality in COVID-19 patients. An age of 60 years or older (OR = 5.05, 95% CI 2.99–8.52), male sex (OR = 2.06, 95% CI 1.42–3.01), and higher CCI (per one-point increase; OR = 1.14, 95% CI 1.06–1.22) were independent risk factors for respiratory failure. Moreover, an age of 60 years or older (OR = 12.02, 95% CI 7.03–20.54), male sex (OR = 1.74, 95% CI 1.31–2.30), CCI (per one-point increase; OR = 1.22, 95% CI 1.16–1.28), and COPD (OR = 1.80, 95% CI 1.11–2.93) were all independent risk factors for all-cause mortality.

**Table 3:** Multivariate analyses of risk factors associated with respiratory failure and all-cause mortality.

Variable	Respiratory failure		All-cause mortality	
	Adjusted OR	95% CI	Adjusted OR	95% CI
Age ≥ 60 years old	5.05	2.99–8.52	12.02	7.03–20.54
Sex, male	2.06	1.42–3.01	1.74	1.31–2.30
CCI, per one point increases	1.14	1.06–1.22	1.22	1.16–1.28
COPD	1.03	0.48–2.25	1.80	1.11–2.93

*CCI* Charlson comorbidity index, *COPD* chronic obstructive pulmonary disease, *OR* odds ratio, *CI* confidence interval.

### Comparison between non-severe and severe COPD groups

Among the COPD patients with COVID-19, characteristics and clinical outcomes were compared between the non-severe and severe groups (Supplementary Tables 2, 3, and 4). The severe group had a higher proportion of older patients (≥ 60 years old) (96.3% vs. 73.7%, *P* = 0.011) than the non-COPD group. The proportions of males, patients with each comorbidity, and patients with each CCI score were similar between the two groups. However, more patients in the severe group showed complete MPRs (51.9% vs. 17.5%, *P* < 0.001), and more patients in the non-severe group had experienced no exacerbations during the measurement period before contracting COVID-19 (85.1% vs. 22.2%, *P* < 0.001) (Supplementary Table 2).

During the COVID-19 period, the total amount of medical resources used was higher in the non-severe group (6264 USD vs. 3585 USD, *P* = 0.003), and none of the patients in the severe group received ICU care or mechanical ventilation. All-cause mortality did not differ between the groups (Supplementary Table 3). According to multivariate analyses of risk factors associated with respiratory failure and all-cause mortality, age (per one year increases; OR = 1.11, 95% CI 1.05–1.17) and male sex (OR = 4.00, 95% CI 1.39–11.55) were significant risk factors for all-cause mortality (Supplementary Table 4).

## Discussion

This is the first nationwide population study to evaluate the impact of COPD on the clinical characteristics and prognosis of COVID-19 patients in South Korea. Greater proportions of COPD patients needed ICU care and mechanical ventilation than of patients without COPD. Among the COVID-19 patients, the risk for all-cause mortality was approximately two times higher in patients with COPD than in those without.

According to our study, among the COVID-19 patients who were 40 years and older, 3.1% had COPD; of these, 7.1% received ICU care, and 5.7% received mechanical ventilation. Among the COVID-19 patients who received ICU care and those who received mechanical ventilation, 5.6% (10/177) and 7.0% (8/116) had COPD, respectively. The rate of all-cause mortality among COPD patients was 19.2%, and the proportion of COPD among COVID-19 non-survivors was 11.9% (27/226). Although COPD was not an independent risk factor for respiratory failure, it was a significant risk factor for all-cause mortality.

Since the World Health Organization declared COVID-19 a pandemic, 79,515,525 cases have been confirmed around the world, with 1,757,947 deaths on January 2nd, 2021^15^. The prevalence of COPD among COVID-19 patients ranges from 0 to 10% worldwide, although most reports are from China, particularly Wuhan or Hubei^3,4,16,17^. In Europe, the prevalence of COPD is between 5.6 and 11%, whereas it is 2.4–5.4% in New York City in the United States^18–25^. However, most of these studies were conducted in a single center or region, resulting in a small population of COVID-19 patients and an even smaller number of COVID-19 patients with COPD^3,5,17^. Therefore, those studies have not evaluated the impact of COPD on the clinical characteristics and prognosis of COVID-19 patients, with the exception of Guan et al. which employed nationwide analyses in China^4^. However, because all COVID-19 patients were reviewed, including those younger than 40 years old, the prevalence of COPD was just 1.5%. Moreover, only 24 patients with COPD were included in the analyses of the impact of COPD on COVID-19^4^.

We have included all COVID-19 patients in South Korea, regardless of whether they were hospitalized or isolated in nonhospital community isolation facilities, termed “living and treatment centers.” Facility admission was initially recommended for patients with asymptomatic or mild COVID-19 cases who were under middle age and had no underlying comorbidities or well-controlled chronic diseases^26^. However, in South Korea, only 6.7% of patients were isolated in living and treatment centers, and 93.3% were hospitalized. In this analysis, we limited the age to 40 years or older to focus on the age group in which COPD is prevalent. In addition, previous studies did not clearly state how the COPD diagnosis was defined, and underestimation of COPD is suspected in overburdened hospitals during the COVID-19 pandemic. We analyzed the HIRA data according to diagnostic codes and medical prescriptions. Our COPD group consisted of patients with significant airway disease who were receiving COPD maintenance medications; thus, COPD patients with asymptomatic mild disease who were not receiving any COPD maintenance medications would have been missed.

Although it is important to keep in mind the small number of COPD patients, it was notable that the proportion of patients with progression to severe COVID-19 among COPD patients has ranged from 20 to 50% in other studies^4–6,27^. This proportion is higher than that reported in our study because our criteria for severe disease were confined to respiratory failure, including the need for mechanical ventilation, ECMO, or ICU care. The rate of all-cause mortality among COPD patients in previous studies ranged from 8.6 to 25%, while the proportion of COPD among COVID-19 non-survivors ranged from 2.8 to 20.0%^1,4,20,28^. During the unexpected COVID-19 pandemic, clinical progression and the outcomes of management vary depending on the status of medical resources and accessibility of medical services in each country. Some countries can provide testing and hospitalization only for patients with severe disease. In South Korea, patients can easily access the hospital, and medical services were provided promptly and equitably to most patients with suspected or confirmed cases of COVID-19.

Increasing age, cardiovascular comorbidities, and high Sequential Organ Failure Assessment scores were significant risk factors for mortality from COVID-19^29^. We also found older age (≥ 60 years old), male sex, and higher CCI to be significant risk factors for respiratory failure and all-cause mortality, as was COPD comorbidity. COPD is a disease that occurs in later life and is associated with multiple comorbidities, including cardiovascular diseases^30,31^. Moreover, COPD itself might have increased the risk for worse clinical outcomes due to poor lung function and immune modulation of the airways. However, COPD is a heterogeneous disease with variation in disease severity, frequency of exacerbations, and comorbidities. Further studies are warranted to determine which features increase the likelihood for poor outcomes in COPD with COVID-19 infection.

This study is the first to describe the impact of the severity of COPD on the clinical course of COVID-19. Our results demonstrate that the severity of COPD does not influence the clinical outcomes of COVID-19, including the length of hospital stay, need for intensive care, respiratory failure, and all-cause mortality. The total amount of medical resources used was higher in the non-severe than in the severe group because a larger absolute number of patients in the non-severe group received intensive care including mechanical ventilation. Among COPD patients, age and male sex were independent risk factors for all-cause mortality. However, because of the small number of patients with severe COPD, the impact of severity could not be thoroughly evaluated, so these results cannot be generalized. Moreover, our strict selection of COPD patients might have failed to identify mild cases with no record of COPD maintenance medication, which could affect the results of comparison between the severe and non-severe COPD groups. However, in a study of the impact of asthma on COVID-19, asthma patients with any previous acute exacerbation before COVID-19 showed mortality twice as high as those without, particularly in case of older age and male sex^32^.

Patients with COPD were more likely to progress to severe disease, leading to a higher mortality rate than in those without COPD. The relationship between COPD and COVID-19 has not been fully elucidated, but several studies have suggested pathways related to ACE-2, to which the spike protein (S protein) of SARS-CoV-2 binds to gain entry into the cell^33,34^. Transmembrane serine protease 2 primes the S protein to facilitate the fusion of the virus with cellular ACE-2 receptors^35^. The expression level of ACE-2 was significantly higher in COPD patients than in controls and in current smokers than in former or never smokers^36–38^. However, the increased expression of ACE-2 in COPD patients would not be sufficient in and of itself to explain the observed increase in susceptibility and increased severity of disease.

The impact of maintenance medication for COPD including ICS, SABA, LABA, and LAMA on the clinical outcomes of COVID-19 infection remain unknown. Among those, ICS has shown controversial results, either mitigating or exacerbating COVID-19 infections^3^. Recently, Schultze et al. reported that regular ICS use in patients with asthma or COPD did not show any beneficial role in protecting against COVID-19-related death, although ICS combinations showed a higher hazard ratio for COVID-19-related death compared to LABA–LAMA combinations, likely due to confounding related to underlying health differences between people who were prescribed ICS and those who were not^39^. Moreover, Choi et al. reported that prior ICS use was not significantly associated with COVID-19 in patients with COPD or asthma, nor with clinical outcomes among patients with COVID-19^40^.

Our study has several potential limitations. First, because the data were obtained from administrative claims, the diagnostic codes and prescribed medications may not have identified all COPD patients, such as those with mild COPD who were not taking COPD maintenance medications. This strict selection of COPD patients might have affected the results of comparison between non-severe and severe groups among COPD patients (Supplementary Tables). Second, due to limitations in obtaining various types of information from the HIRA database, any details of medical procedures were not available and only few variables were adjusted in regression analyses. For example, information on smoking status was not available in the HIRA data, and smoking may be associated with the expression of ACE-2 in COPD patients. Third, further detailed information about inhalers used as maintenance medication to control COPD and the amount of systemic corticosteroids were not analyzed with regard to their effects on the risk for and progression of COVID-19. Moreover, although we analyzed the number of previous exacerbations in COPD patients before they contracted COVID-19, the impacts of exacerbations were not fully evaluated because of the small number of COPD patients.

In conclusion, COPD is an independent risk factor for all-cause mortality in COVID-19 patients in Korea. In addition, older age, male sex, and a higher CCI score were significant risk factors for respiratory failure and all-cause mortality. Although the severity of COPD was not associated with clinical outcomes in COVID-19 patients, further research is needed to investigate the association in a larger group of patients. Our study suggests that close attention and special consideration with regard to COPD patients are essential to prevent the progression of COVID-19, leading to mortality.

## Supplementary Information

Supplementary Information

## Data Availability

The datasets used and/or analyzed in the current study are not publicly available due to patient data privacy regulations pertaining to HIRA.
