# Supplementary material for: Impact of COPD on COVID-19 prognosis: A nationwide population-based study in South Korea
Source: Sci Rep. 2021 Feb 12;11:3735. doi: 10.1038/s41598-021-83226-9 (PMC7880985; doi:10.1038/s41598-021-83226-9)
Supplement: Supplementary file 1 — Supplementary Information [file 41598_2021_83226_MOESM1_ESM.docx]

**Supplementary Materials**

**Impact of COPD on COVID-19 prognosis: A nationwide population-based study in South Korea**

Sang Chul Lee, MD; Kang Ju Son, Master’s degree; Chang Hoon Han, MD; Seon Cheol Park, MD; Ji Ye Jung, MD, PhD

**Supplementary Table 1. Determinants of the Charlson Comorbidity Index**

| **Score** | **Condition** |
| --- | --- |
| 1 | Myocardial infarction |
| 1 | Congestive heart failure |
| 1 | Peripheral vascular disease |
| 1 | Cerebrovascular disease |
| 1 | Dementia |
| 1 | Chronic pulmonary disease |
| 1 | Connective tissue disease |
| 1 | Peptic ulcer disease |
| 1 | Mild liver disease |
| 2 | Hemiplegia |
| 2 | Moderate or severe renal disease |
| 2 | Diabetes with end-organ damage |
| 2 | Any malignancy without metastasis |
| 2 | Leukemia |
| 2 | Lymphoma |
| 3 | Moderate or severe liver disease |
| 6 | Metastatic solid malignancy |
| 6 | Acquired immune deficiency syndrome |

**Supplementary Table 2. Characteristics of COVID-19 patients with COPD according to the different severity of COPD**

| **Characteristics** | **Non-severe**  **(n = 114)** | **Severe**  **(n = 27)** | ***P*-value** |
| --- | --- | --- | --- |
|  |  |  |  |
| Age, years |  |  |  |
| 40-59 | 30 (26.3) | 1 (3.7) | 0.011 |
| ≥ 60 | 84 (73.7) | 26 (96.3) |  |
| Sex, male | 56 (51.8) | 15 (55.6) | 0.722 |
| Comorbidities |  |  |  |
| Hypertension | 67 (58.8) | 18 (66.7) | 0.451 |
| Diabetes | 59 (51.8) | 15 (55.6) | 0.722 |
| Ischemic heart disease | 27 (23.7) | 6 (22.2) | 0.872 |
| Angina pectoris | 23 (20.2) | 5 (18.5) | 0.846 |
| Myocardial infarction | 7 (6.1) | 1 (3.7) | 0.623 |
| Heart failure | 33 (28.9) | 6 (22.2) | 0.482 |
| Cerebrovascular disease | 28 (24.6) | 10 (37.0) | 0.189 |
| Rheumatological disease | 10 (8.8) | 2 (7.4) | 0.819 |
| Liver disease | 9 (7.9) | 2 (7.4) | 0.932 |
| Malignancies | 17 (14.9) | 3 (11.1) | 0.611 |
| CCI score |  |  |  |
| 0-1 | 13 (11.4) | 0 (0) | 0.169 |
| 2 | 22 (19.3) | 8 (29.6) |  |
| 3 | 10 (8.8) | 1 (3.7) |  |
| ≥ 4 | 69 (60.5) | 18 (66.7) |  |
| MPR group^*^ |  |  |  |
| Low | 91 (79.8) | 11 (40.7) | < 0.001 |
| Partial | 3 (2.6) | 2 (7.4) |  |
| Complete | 20 (17.5) | 14 (51.9) |  |
| Number of exacerbations^†^ |  |  |  |
| 0 | 97 (85.1) | 6 (22.2) | <0.001 |
| 1 | 17 (14.9) | 3 (11.1) |  |
| ≥2 | 0 (0) | 18 (66.7) |  |

Notes: Data are presented as numbers (%).

* MPR was calculated as the sum of all days’ supplies of prescriptions filled divided by the time from the first filling of a prescription until the end of the measurement period. Patients were categorized into three adherence groups: low (MPR <0.5), partial (MPR 0.5–0.79), and complete (MPR ≥0.8) adherence.

† Exacerbation was defined when the diagnostic code for COPD (J43 and J44 except J43.0) was present in conjunction with any of the following: (1) treatment with an antibiotic or systemic corticosteroids, (2) hospitalization, or (3) an emergency room visit.

COPD, chronic obstructive pulmonary disease; CCI, Charlson Comorbidity Index; MPR, medication possession ratio.

**Supplementary Table 3. Clinical outcomes of COVID-19 in COPD patients according to the different severity of COPD**

| **Variables** | **Non-severe**  **(n = 114)** | **Severe**  **(n = 27)** | ***P*-value** |
| --- | --- | --- | --- |
|  |  |  |  |
| Total amount of medical resource use, USD | 6,264 ± 7,257 | 3,585 ± 2,877 | 0.003 |
| Hospital admission |  |  |  |
| Number of patients | 108 (94.7) | 26 (96.3) | 0.737 |
| Length of stay, days | 26.3 ± 19.0 | 24.2 ± 19.4 | 0.619 |
| ICU care |  |  |  |
| Number of patients | 10 (8.8) | 0 (0) | 0.110 |
| Length of stay, days | 9.7 ± 8.8 | - | - |
| Mechanical ventilator | 8 (7.0) | 0 (0) | 0.156 |
| ECMO | 0 (0) | 0 (0) | - |
| All-cause mortality | 22 (19.3) | 5 (18.5) | 0.926 |

Notes: Data are presented as numbers (%) or means ± standard deviations.

COVID-19, coronavirus disease 2019; COPD, chronic obstructive pulmonary disease; USD, United States dollar; ICU, intensive care unit; ECMO, extracorporeal membrane oxygenation.

**Supplementary Table 4 Multivariate analyses of risk factors associated with respiratory failure and all-cause mortality in COPD patients**

| **Variable** | **Respiratory failure risk** | | **All-cause mortality** | |
| --- | --- | --- | --- | --- |
|  | **Adjusted OR** | **95% CI** | **Adjusted OR** | **95% CI** |
| Age, per one year increase | 1.02 | 0.97–1.09 | 1.11 | 1.05–1.17 |
| Sex, male | 2.73 | 0.51–14.5 | 4.00 | 1.39–11.55 |
| Severity: severe (vs. non-severe)* | - | - | 0.82 | 0.19–3.39 |
| MPR: partial/complete (vs. low)† | - | - | 0.70 | 0.23–2.12 |
| # of exacerbations: ≥ 1 (vs. 0)^§^ | 0.91 | 0.09–8.75 | 1.22 | 0.36–4.13 |

* Severe COPD included who had experienced exacerbations two or more times, or those who had been prescribed triple therapy (ICS, LABA, and LAMA), PDE-4 inhibitors or low-dose macrolides.

† MPR was calculated as the sum of all days’ supplies of prescriptions filled divided by the time from the first filling of a prescription until the end of the measurement period. Patients were categorized into three adherence groups: low (MPR <0.5), partial (MPR 0.5–0.79), and complete (MPR ≥0.8) adherence.

^§^ Exacerbation was defined when the diagnostic code for COPD (J43 and J44 except J43.0) was present in conjunction with any of the following: (1) treatment with an antibiotic or systemic corticosteroids, (2) hospitalization, or (3) an emergency room visit.

COPD, chronic obstructive pulmonary disease; OR, odds ratio; CI, confidence interval
